# Preliminary experience in treating thoracic spinal tuberculosis via a posterior modified transfacet debridement, instrumentation, and interbody fusion

**DOI:** 10.1186/s13018-018-0994-8

**Published:** 2018-11-20

**Authors:** Yun-Peng Huang, Jian-Hua Lin, Xiao-Ping Chen, Gui Wu, Xuan-Wei Chen

**Affiliations:** 1 Department of Spine Surgery, The First Affiliated Hospital of Fujian Medical University, 20 Chazhong Road, Fuzhou City, 350005 Fujian Province China; 2 School of Mathematics and Informatics, Fujian Normal University, Fuzhou City, 350117 Fujian Province China

**Keywords:** Focal debridement, Modified transfacet approach, Surgical treatment, Thoracic spine tuberculosis

## Abstract

**Background:**

Posterior transfacet approach has been proved to be a safe and effective access to treat thoracic disc herniation. However, the therapeutic effect and safety of modified transfacet approach for treating thoracic spinal tuberculosis (TST) has not been reported in the clinical literature. In this study, the clinical efficacy and safety of a single-stage posterior modified transfacet debridement, posterior instrumentation, and interbody fusion for treating TST were retrospectively evaluated.

**Patients and methods:**

From 2009 to 2014, 37 patients with TST underwent a posterior modified transfacet debridement, interbody fusion following posterior instrumentation, under the cover of 18 months of antituberculosis chemotherapy. The patients were evaluated preoperatively and postoperatively in terms of Frankel Grade, visual analog scale (VAS) pain score, kyphotic Cobb angle, and bony fusion.

**Results:**

The follow-up time was 39.8 ± 5.1 months (29–50 months). No postoperative complication or recurrence of spinal tuberculosis was observed. Definitive bony fusion was achieved in all patients. At the final follow-up, 2 cases were rated as Frankel grade D, 35 as grade E. VAS was recovered from 8.4 ± 1.0 cm to 0.4 ± 0.8 cm. The kyphotic angles were corrected from 29.4 ± 10.9° to 17.6 ± 6.3°. Using the Kirkaldy-Willis criteria, functional outcome was excellent in 29 patients, good in 7, and fair in 1.

**Conclusions:**

Our preliminary results showed that single-stage posterior modified transfacet debridement, posterior instrumentation, and interbody fusion are effective and safe surgical options for treating TST.

## Background

The incidence of tuberculosis (TB) is rising. In China, spinal TB is the most common form of extrapulmonary tuberculosis and remains a severe public health threat. According to the World Health Organization, 1.4 million new cases of TB occur annually in China, with spinal TB found in approximately 1% of all affected patients [1]. Thoracic spine tuberculosis (TST) is the most common spinal tuberculosis (TB), leading to local pain, paralysis, kyphotic deformity, and even death [2]. Although antituberculosis chemotherapy is the mainstay in the management of the disease, surgical treatment is indicated for patients with cold abscess, neurologic lesion, spinal instability, kyphosis, and/or failure of conservative treatment [3]. The primary goals of a surgical approach are to completely debride the lesion, restore nerve function, and correct and avoid spinal deformity progression. Various surgical approaches have been developed, including anterior, posterior, and combined antero-posterior approaches. Selection of the optimum surgical approach remains controversial [4–6]. Anterior or combined antero-posterior approaches are associated with a high rate of major mobility and mortality [4, 7, 8]. On the other hand, posterior approaches such as posterolateral [3], transpedicular [9], or the transforaminal approach [7] have been favored due to their simple anatomical demands and a lower rate of complications. However, even these approaches require a relatively extensive bone dissection and tissue disruption to provide adequate exposure of the lesion via removal of the proximal rib, costotransverse articulations, and posterior elements (including spinous process, lamina, facet joints and transverse process) linked to complications including pneumothorax and postoperative pain, particularly in multilevel TST [3, 7, 10].

The posterior transfacet approach and its variations have been shown to be a safe and effective method for the treatment of thoracic disc herniation (TDH) with relatively low morbidity [11, 12]. Furthermore, the posterior approach can provide a better correction of the kyphosis which most frequently occurs with TST. Anatomically, there is lesion of the intervertebral disk space in TST [13, 14], which is similar to that in TDH. Therefore, the authors believe that TST patients can be managed adequately by the posterior modified transfacet approach. The aim of this study was to validate the efficacy and safety of posterior modified transfacet debridement, instrumentation, and interbody fusion for the treatment of TST.

## Methods

This study was approved by the Ethics Committee of the Hospital. Between 2009 and 2014, the authors treated 37 consecutive patients with TST via a modified transfacet approach. All patients were treated by the same surgical team though the team treated other patients (non-participants) with other approaches during the study period. The cohort was comprised of 22 males and 15 females, with an average age of 43.1 ± 17.3 years. The diagnosis of TST was based on clinical presentation, plain radiographs, computed tomography (CT), magnetic resonance imaging (MRI), hematologic tests, and pathological examinations from CT-guided biopsy. Twenty-three were confirmed radiologically as multilevel TST. Of these, 5 were non-contiguous multifocal TST. Twenty-five were accompanied with paraspinal abscess. Eleven cases had bilateral paraspinal abscess and 4 had large abscess. One patient had abdominal draining sinuses before surgery. Twenty-six patients had a neurological deficit of grade B, C, or D according to the Frankel Grade, including motor weakness, sensory change, and radiating pain to the lower limbs. Eleven cases had combined pulmonary tuberculosis. Patients rated their pain intensity on a visual analog scale (VAS), from “no pain” (0 cm) to “maximal pain” (10 cm). The mean preoperative VAS score of the cohort was 8.4 ± 1.0 cm (range 5.4–9.8 cm).

### Preoperative procedure

Patients were treated with the standard regimen of isoniazid (H), rifampin (R), ethambutol (E), and pyrazinamide (Z) (HREZ) chemotherapy regimen [11], consisting of isoniazid (300 mg/d), rifampicin (450 mg/d), ethambutol (750 mg/d), and pyrazinamide (750 mg/d) for at least 4 weeks before surgery. The erythrocyte sedimentation rate (ESR) was 44.7 ± 23.3 mm/h; the kyphosis angle was 29.4 ± 10.9°. When the ESR had significantly decreased (< 40 mm/h), surgery was carried out. The preoperative anti-TB treatment reduces *Mycobacterium tuberculosis* in lesions and increases surgical safety [15]. Preoperative clinical and radiological characteristics are shown in Tables 1 and 2.

**Table 1:** Patient demographics, operative information, and disease characteristics

No.	Gender	Age (years)	Levels	Focal debridement	Operative time (min)	Blood loss (ml)	Follow-up (months)	Bone fusion (months)	Complications
1	F	39	T10–11	T10–11	210	500	42	6	
2	F	62	T7–9	T7–8	230	300	45	9	
3	M	52	T5–6.	T5–6	208	200	36	6	
4	F	75	T5–7	T5–6	230	700	47	12	
5	M	65	T6–7	T6–7	220	250	45	6	
6	M	27	T11–L1	T11–12	160	200	36	6	Pain at the donor site
7	F	39	T11–12	T11–12	180	300	39	6	
8	M	48	T1–2, T5–6	T5–6	230	300	35	6	
9	M	64	T10–12	T10–11, T11–12	290	600	38	9	
10	M	64	T8–9	T8–9	215	400	38	6	
11	F	24	T4–L2	T6–7, T11–12	410	1800	29	12	Hypoproteinemia
12	F	81	T10–11	T10–11	240	500	40	6	
13	F	59	T8–9	T8–9	152	100	45	6	
14	F	39	T10–L2	T10–11	230	450	43	6	
15	M	31	T11–L1	T11–12	200	400	36	6	
16	M	64	T10–12	T10–11	195	600	39	6	
17	F	76	T8–9	T8–9	202	500	37	6	
18	M	24	T8–L2	T9–10, T11–12	310	800	40	9	
19	M	55	T10–L2	T11–12	230	300	33	6	
20	M	21	T4–12	T6–7, T9–10	360	1500	43	9	Water–electrolyte imbalance
21	M	33	T6–L1	T8–9, T11–12	320	1000	48	9	
22	F	40	T10–L2	T11–12	220	450	33	6	
23	F	23	T8–L3	T9–10, T12–L1	355	1300	41	12	
24	M	30	T8–L1	T9–10	190	300	48	6	
25	M	36	T11–L2	T11–12	210	700	42	6	
26	M	23	T10–L4	T10–11	180	200	50	6	
27	M	28	T11–L1	T11–12	110	200	31	6	
28	M	42	T8–10	T8–9	270	500	40	6	
29	M	39	T7–9	T8–9	160	300	36	6	
30	M	31	T7–L1	T9–10, T10–11	240	600	37	6	
31	M	26	T5–11	T6–7, T9–10	290	1200	46	9	
32	M	44	T6–7	T6–7	190	200	40	6	
33	F	60	T8–9	T8–9	180	300	42	9	Pain at the donor site
34	M	23	T5–6	T5–6	200	350	33	6	
35	F	48	T9–10	T9–10	130	200	38	6	
36	F	25	T7–8	T7–8	170	300	35	6	
37	F	34	T6–8	T7–8	160	300	46	6	

*M* male, *F* female, *T* thoracic spine, *L* lumbar spine

**Table 2:** Summary of clinical and radiological data

No.	Kyphosis (°)			Frankel grade			ESR (mm/h)			VAS (mm)		Kirkaldy-Willis criteria
	Preop	Postop	FFU	Preop	Postop	FFU	Preop	Postop	FFU	Preop	FFU	
1	19.5	13.7	14.5	E	E	E	9	12	10	9.8	0	Excellent
2	38.3	28.8	30.1	D	D	E	49	7	6	8.9	0	Excellent
3	23.7	15.9	16.3	E	E	E	26	11	10	7.8	0	Excellent
4	23.2	17.3	19.1	D	D	E	40	10	7	8.3	0	Excellent
5	25.4	16.7	18.0	E	E	E	117	36	10	9.1	0	Excellent
6	13.7	6.1	7.2	E	E	E	95	22	4	6.8	2	Good
7	11.9	9.1	9.2	E	E	E	51	13	11	8.4	0	Excellent
8	35.7	27.7	29.5	B	C	D	26	9	7	8.9	3.5	Fair
9	25.2	17.5	19.1	E	E	E	16	11	12	7.5	0	Excellent
10	17.6	9.8	9.8	C	D	D	67	15	5	7.7	1	Good
11	38.2	35.1	35.1	E	E	E	52	19	7	7.4	0	Excellent
12	15.9	11.7	12.0	C	E	E	19	6	8	6.3	0	Excellent
13	20.2	16.3	16.9	D	D	E	45	12	9	8.2	0	Excellent
14	17.0	9.2	9.0	E	E	E	83	14	11	9.0	0	Excellent
15	29.3	17.5	20.1	C	E	E	32	12	9	8.6	0	Excellent
16	27.2	18.8	19.2	D	E	E	36	9	8	9.3	0	Excellent
17	32.5	20.2	19.7	C	E	E	26	12	15	7.6	1	Good
18	25.5	12.5	13.2	D	E	E	79	15	7	9.1	0	Excellent
19	28.3	17.7	18.4	D	E	E	55	9	11	8.4	0	Excellent
20	49.0	23.6	24.3	D	E	E	47	12	15	7.8	0	Excellent
21	45.8	21.3	23.6	C	D	E	87	25	12	9.0	2	Good
22	26.3	13.4	14.5	D	E	E	39	15	14	8.2	0	Excellent
23	33.1	19.5	20.7	D	D	E	24	13	9	9.3	0	Excellent
24	17.9	7.1	9.8	E	E	E	33	7	10	8.8	0	Excellent
25	31.4	18.8	19.4	D	E	E	45	12	9	9.6	2.6	Good
26	28.3	13.4	14.2	D	E	E	23	10	13	9.4	0	Excellent
27	22.0	10.4	11.6	E	E	E	51	11	9	7.8	0	Excellent
28	37.5	19.5	19.7	B	D	E	18	10	10	5.4	1	Good
29	39.7	18.6	19.3	E	E	E	43	10	7	9.7	0	Excellent
30	36.3	20.4	21.7	C	D	E	30	19	10	7.9	0	Excellent
31	65.4	23.4	26.4	B	C	E	51	20	13	8.8	1	Good
32	36.5	16.3	19.1	D	E	E	49	21	9	8.2	0	Excellent
33	28.2	8.8	10.9	C	D	E	39	9	9	8.0	0	Excellent
34	30.4	13.9	17.4	D	E	E	41	12	10	9.5	0	Excellent
35	17.5	9.9	10.7	D	E	E	26	8	6	7.6	0	Excellent
36	33.1	11.3	13.6	C	D	E	47	10	10	7.9	0	Excellent
37	42.4	14.6	17.3	D	E	E	38	9	7	9.4	0	Excellent

The reference value of ESR in our hospital is as follows: < 20 mm/h (male), < 15 mm/h (female)

*Preop* preoperative, *Postop* postoperative 3 months, *FFU* final follow-up, *ESR* erythrocyte sedimentation rate, *VAS* visual analog scale

### Surgical technique

**Fig. 1:**
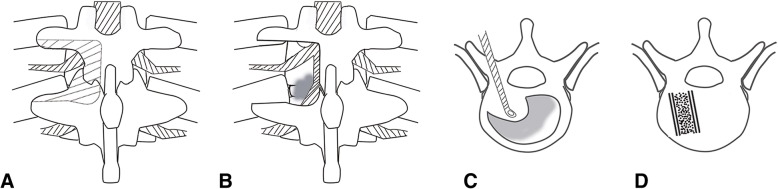
Posterior modified transfacet debridement and fusion (developmental view). A facetectomy and a partial hemi-laminectomy are performed to expose the dura, disc space, and granulation tissue (**a**, **b**). Intervertebral focal debridement (**c**). Autogenous bone in lesions after debridement (**d**)

The modified transfacet approach was similar to that described by Bransford et al. [12] for approaching protruded thoracic discs. Under general anesthesia, patients were placed in prone position and a linear, midline incision was made. The spinous process, lamina, facet joints, and transverse processes were exposed (subperiosteal dissection). Pedicle screws were inserted at least two levels above and below the level of involvement (outlined in Table 1). Pedicle screws were placed in the affected vertebrae if the vertebrae were not destroyed by infection. For thoracic spine, a unilateral or bilateral facet complex and parts of the lamina were removed, exposing the affected disc space (lateral one-third), granulation tissue, lateral aspect of dural sac, and exiting nerve root. When required, the upper and lower transverse processes were partially excised to increase the exposure (Fig. 1a, b). No rib or thoracic nerve root was removed in any patient. When there was a large paraspinal abscess, a catheter was inserted deep into the abscess cavity to flush the abscess until no pus outflowed. The annulus was opened with a blade, and debridement in the affected intervertebral space was subsequently performed with rongeurs and curettes until the sclerotic bone, disc, pus, and granulation tissue were completely removed through to healthy bleeding bone (working in a lateral to medial direction to create a central cavity). Granulation tissue adherent to the ventral aspect of the dural tube was pushed downward into the central cavity with curettes and was then removed piecemeal (Fig. 1c). Anterior spinal cord decompression was obtained. Multi-affected intervertebral spaces were chosen for focal debridement if there was involvement of a long segment [4]. Tricortical harvested from the iliac crest was implanted for posterior fusion in the intervertebral space that underwent focal debridement (Fig. 1d). Two pre-bent titanium rods were fixed to correct the local deformity, and screws were compressed to achieve bone-to-bone contact at the anterior column. Finally, after irrigation by sterile physiologic saline, 0.5 g streptomycin was embedded in the pathological intervertebral space [3]. Two drainage tubes were inserted routinely, and the incision was closed. The material debrided was sent for culturing and histopathologic examination.

### Postoperative procedure

The postural drain was usually removed when drainage volume is < 50 ml/24 h. For patients with large paraspinal abscess, percutaneous drainage was performed under sonographic or CT guidance [16]. Patients continued with the oral HREZ chemotherapy post-operatively. Six months later, pyrazinamide was discontinued. Patients then received a 12-month regimen of HRE chemotherapy [11]. Frankel Grade, VAS, and ESR were evaluated monthly, and X-ray was examined every 3–6 months. CT and/or MRI scan were taken at the 18-month or final follow-up (Table 1). Bony spinal fusion was assessed according to the criteria defined by Lee et al. [6, 17]. Functional outcome was assessed by Frankel Grade and the Kirkaldy-Willis criteria [18]. Using SPSS 19.0 software (SPSS, Inc., Chicago, IL, USA), VAS, ESR, Frankel Grade, and kyphosis angles were statistically analyzed by paired *t* test pre- and post-operatively and final follow-up. Using R software (version 3.2.2, 2015), the *P* values of paired *t* test were adjusted for multiple comparison by BH (Benjamini & Hochberg, 1995) method [19]. *P* < 0.05 was considered significant.

## Results

**Fig. 2:**
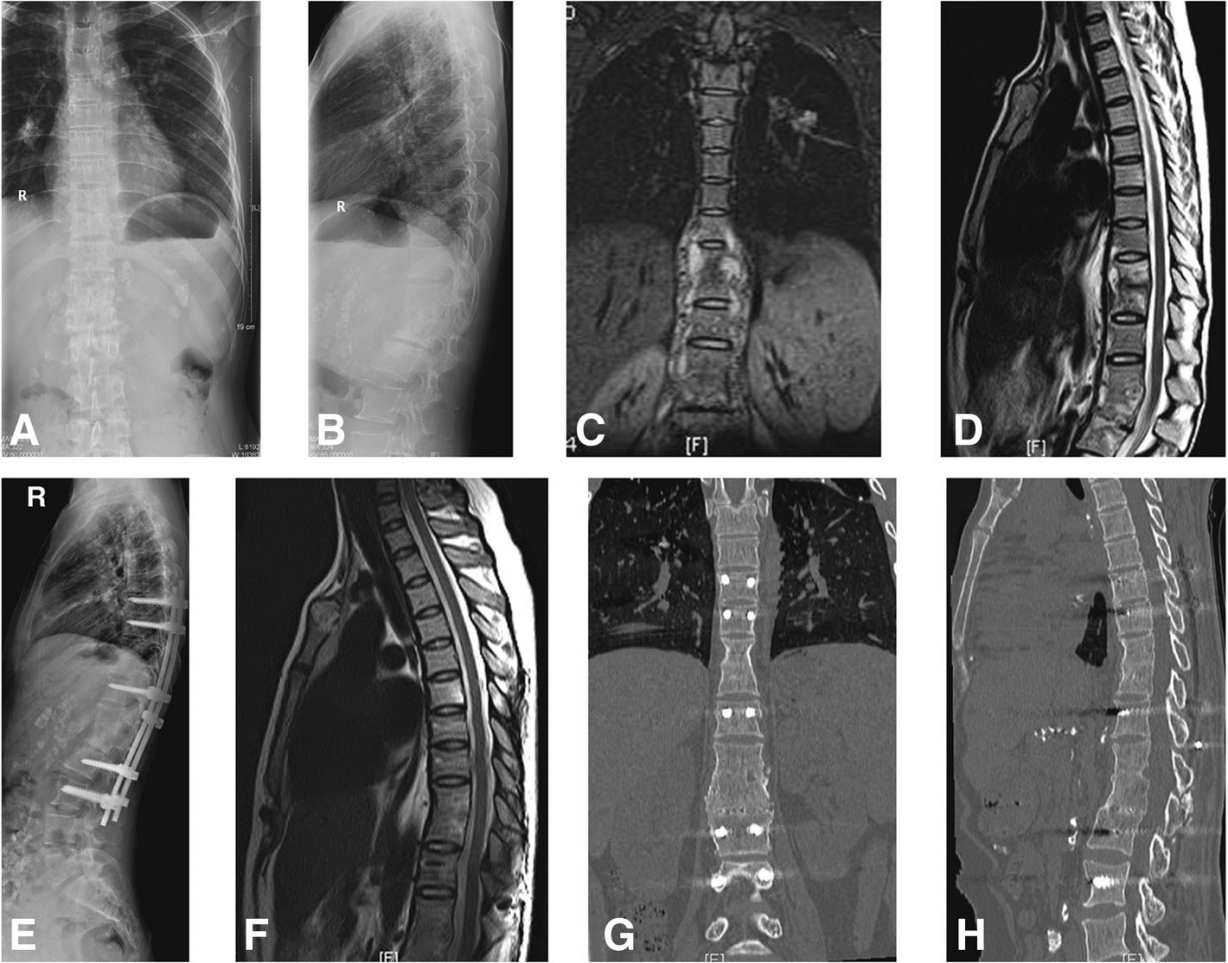
Preoperative radiography (**a**, **b**) and MRI (**c**, **d**) of a 23-year-old female patient with tuberculosis at T8–L3 with extensive paravertebral abscesses. Radiography (**e**), MRI (**f**), and CT (**g** and **h**) at the final follow-up showed definitive interbody bone fusion was achieved at T9–10 and T12–L1. MRI: magnetic resonance image; CT: computed tomography scan

**Fig. 3:**
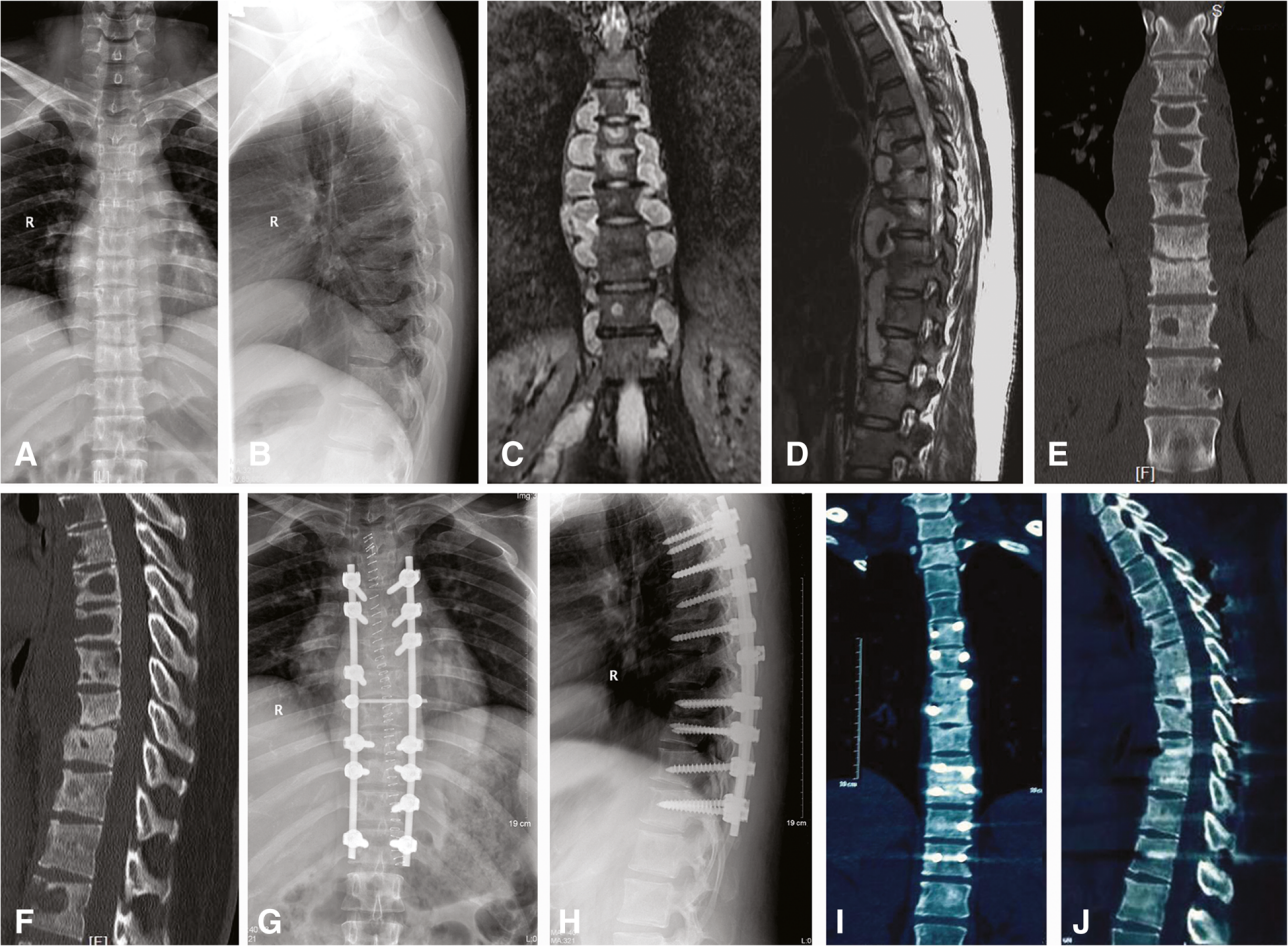
Preoperative radiography (**a** and **b**), MRI (**c** and **d**), and CT (**e** and **f**) of a 24-year-old female patient with tuberculosis at T4–L2 with extensive paravertebral abscesses. Radiography (**g**, **h**) and CT (**i**, **j**) at the final follow-up showed definitive interbody bone fusion was achieved at T6–7 and T9–10 (**e**–**h**). MRI: magnetic resonance image; CT: computed tomography scan (**i**–**j**)

For all cases, the mean follow-up time was 39.8 ± 5.1 months (29–50 months). The mean operative time, blood loss, and duration of hospital stay were presented in Table 1. No severe operation-related complications including sinus formation, dural tear, wound infection, or pneumothorax were observed (Table 2). During the period, no clinical or radiological relapse was found. At the final follow-up, all patients showed satisfactory clinical, laboratory, and imaging basis eradication of the infection. All patients achieve definitive bony fusion with an average time of 7.1 ± 1.9 months [17]. The average VAS pain score dropped to 0.4 ± 0.8 cm (range 0.0–3.5 cm) at the final follow-up (*P* < 0.01). ESR returned to normal (13.2 ± 5.9 mm/h) within 3 months after surgery (*P* < 0.01). The mean kyphotic angle before and after surgery and at the final follow-up was 29.4 ± 10.9°, 16.4 ± 6.2°, and 17.6 ± 6.3°, respectively. The pre- and post-operative differences were statistically significant (*P* < 0.01), as were the post-operative and final (*P* < 0.01) Cobb angles. At the final follow-up visit, the neurologic status of 2 patients with preoperative neurologic deficit improved by three grades, 7 by two grades, and 16 by one grade (Table 2). Using the Kirkaldy-Willis criteria [18], functional outcomes were denoted as excellent in 29 patients, good in 7, and fair in 1. The typical cases are shown in Figs. 2 and 3.

## Discussions

TST accounts for the largest proportion (30.3–55.8%) of spinal tuberculosis cases [2]. Anti-TB chemotherapy and surgery are currently the standard methods for treating TST. Surgical approaches for TST have evolved over time, including anterior, posterior, and combined antero-posterior approaches. Anterior and combined approaches are often associated with higher morbidities and mortality, although they grant direct access to debridement and strut grafts [20]. Conversely, the posterior approach is simple and associated with a low risk of morbidity [7, 10]. However, the posterior approach for TST has its own risk profile, owing to its special anatomical characteristics and positioning. For most cases of TST, the anterior and middle columns are difficult to operate from the back. On the other hand, the thoracic spinal canal allows little room for intraoperative manipulation and the spinal cord is vulnerable to damage. Therefore, the posterior approach and its variants require proximal rib and costotransverse articulation resection in order to offer direct exposure to lateral aspects of the diseased vertebral bodies [3, 10]. Additionally, total laminectomy is required to achieve decompression. In sum, the complications of posterior surgery are not minimal and are usually related to the surgical outcomes.

A simpler operation with fewer risks is desirable, especially for the high-risk patient. A posterior transfacet approach was described initially by Stillerman et al. [11] and modified by other authors [12, 21]. This procedure for the treatment of TDH has yielded excellent results. The essence of the transfacet approach is to provide safe and effective access for the removal of herniated discs with relatively low morbidity [11, 22]. The anatomical characteristics of TDH are similar to those of TST since *Mycobacterium tuberculosis* is also prone to affect the anterior column including the intervertebral disc and its upper and lower adjoining vertebral bodies (peridiscal) [14, 23]. In light of this, we used a posterior transfacet approach for the treatment of TST, as described for the treatment for TDH. In this study, we modified this approach to allow for the safe debridement and interbody fusion via complete facetectomy and partial hemilaminectomy to minimize tissue disruption and preserve ribs and the partial posterior element of the thoracic spine [12]. In our study, all patients obtained satisfactory results with respect to pain relief, neurologic function, kyphosis correction, bone fusion, and laboratory findings. No relapse was detected in our patients by the time of the last follow-up visit.

Compared with conventional posterior approaches, our approach has a limited view. Clinically, our preliminary experience showed that the posterior modified transfacet approach provides an adequate exposure and acceptable access for effective focal debridement. The reasons may be attributed to the following. First, resection of one or both sides of thoracic facet joint and partial hemi-laminectomy offers an oblique visualization and posterolateral manipulations facilitate effective focal debridement in the affected intervertebral space and limit “around-the-corner” or blind-spot dissection [12]. Additionally, partial removal of the upper and lower transverse processes enlarges the exposure to focal lesions and increases the window for posterolateral manipulation. Third, during focal debridement, the sclerotic bone, dead osteons, pus, granulation tissue, and disc were completely removed, reaching the subnormal substance of bones between normal cancellous bones and pathologic bones [3, 5, 24, 25]. Fourth, for multilevel TST, the affected foci were chosen to perform debridement separately since it is unnecessary to achieve debridement in each lesion as radical debridement is relative in any surgical approach [3, 5, 9]. Finally, anti-TB chemotherapy, rest, and nutritional improvement are still the most basic methods of TB treatment [3, 13].

The posterior approach has become popular, but potential operation-related complications and morbidity remain a serious concern. Luo et.al [26] described a pneumonia rate of 16.2%, a cerebrospinal fluid (CSF) leakage rate of 5.4%, and a thrombosis rate of 2.7% in 37 cases treated with a posterior transpedicular approach. Yin et.al [3] reported that 5 of 31 patients operated via a posterior approach with costotransversectomy experienced complications including pneumothoraxic, CSF leakage, sinus formation, and wound infection. The purpose of posterior modified transfacet approach is minimizing the surgical impact. Owing to its simple anatomy, this approach minimizes the intraoperative and postoperative complications that may occur with the posterolateral, transpedicular, or transforaminal approach. In our study, no severe complication was noted.

The following are the major advantages of this approach. First, posterolateral manipulation during focal debridement produces a “cavitation” of the intervertebral space to allow the granulation tissue be completely pushed without any retraction of the dura sac [12], which minimizes the risks of dura tears, CSF leakages, or worsening of neurological deficiencies. Second, this approach allows for simultaneous debridement and stabilization of the spine via a single posterior midline incision, compared with the posterolateral [3] or transforaminal approaches [7]. Third, bone removal and soft-tissue disruption are minimal since the modified transfacet approach obviates the need for dissecting the proximal rib and exposing the lateral aspects of the infected vertebral bodies. Thus, it eliminates the risk of pneumothoraxic complications and diminishes long-term localized pain. Fourth, compared with total laminectomy, facetectomy and partial hemilaminectomy have been reported to lead to reduced operation time, bone loss, and tissue disruption, especially for patients with multilevel disease. Lastly, a single posterior midline incision and the minimal amount of bone removal and tissue dissection result in decreased intraoperative anesthetic, shorter operative time, less blood loss, shorter hospital stays, and less time off normal activities. Due to the small sample and single site study, the above findings should be validated in expanded and comparative method studies.

The main limitation to this study was the relatively small series of patients enrolled in a single institution. Nonetheless, patient data continues to be accumulated including clinical follow-up of patients. Additionally, no comparative treatment group was available to the transfacet approach such as anterior, posterior, or combined anterior and other posterior approaches. Finally, the transfacet approach has its potential limits which should be comprehensively discussed. All things considered, the author considers that the following indications are inappropriate for this approach: (1) lesions confined to the anterior vertebral column, (2) > 50% collapse of the vertebral body, and (3) severe segment kyphosis.

## Conclusions

Our experience suggests that posterior modified transfacet approach for the treatment of TST is safe and effective, providing adequate exposure for intervertebral focal debridement, posterior instrumentations, and interbody fusion. The results using this technique were excellent. The risk of operation-related complications was minimized, likely owing to the simple anatomy, minimal bone dissection, and tissue disruption of the technique. This approach may become the procedure of choice in the surgical management of all TST. Future studies should attempt to reproduce the results of this study with larger number of patients and longer follow-up times.

## Abbreviations

ASIA: The American Spinal Injury Association
CSF: Cerebrospinal fluid
CT: Computed tomography
ESR: Erythrocyte sedimentation rate
FFU: Final follow-up
HREZ: The standard regimen of isoniazid (H), rifampin (R), ethambutol (E), and pyrazinamide (Z)
MRI: Magnetic resonance imaging
Postop: Postoperative
Preop: Preoperative
TB: Tuberculosis
TDH: Thoracic disc herniation
TST: Thoracic spine tuberculosis
VAS: Visual analog scale
